# Gray and white matter abnormalities in primary trigeminal neuralgia with and without neurovascular compression

**DOI:** 10.1186/s10194-020-01205-3

**Published:** 2020-11-25

**Authors:** Min Wu, Xiaofeng Jiang, Jun Qiu, Xianming Fu, Chaoshi Niu

**Affiliations:** 1 Department of Neurosurgery, The First Affiliated Hospital of USTC (Anhui Provincial Hospital), Division of Life Sciences and Medicine, University of Science and Technology of China, Hefei, Anhui 230001 P.R. China; 2 Anhui Provincial Stereotactic Neurosurgical Institute, Hefei, Anhui 230001 P.R. China; 3 Department of Diagnostic Radiology, The First Affiliated Hospital of USTC (Anhui Provincial Hospital), Division of Life Sciences and Medicine, University of Science and Technology of China, Hefei, Anhui 230001 P.R. China

**Keywords:** Trigeminal neuralgia, Voxel-based morphometry, Surface-based morphometry, Diffusion tensor imaging, Internal neurolysis, Neurovascular compression, Conditioned pain modulation

## Abstract

**Purpose:**

Previous researches have reported gray and white matter microalterations in primary trigeminal neuralgia (TN) with neurovascular compression (NVC). The central mechanism underlying TN without NVC are unknown but may include changes in specific brain regions or circuitries. This study aimed to investigate abnormalities in the gray matter (GM) and white matter (WM) of the whole brain and the possible pathogenetic mechanism underlying this disease.

**Methods:**

We analyzed brain morphologic images of TN patients, 23 with NVC (TN wNVC) and 22 without NVC (TN wNVC) compared with 45 healthy controls (HC). All subjects underwent 3T-magnetic resonance imaging and pain scale evaluation. Voxel-based morphometry (VBM) and surface-based morphometry (SBM) were used to investigate whole brain grey matter quantitatively; graph theory was applied to obtain network measures based on extracted DTI data based on DTI data of the whole brains. Sensory and affective pain rating indices were assessed using the visual analog scale (VAS) and short-form McGill Pain Questionnaire (SF-MPQ).

**Results:**

The VBM and SBM analyses revealed widespread decreases in GM volume and cortical thickness in TN wNVC compared to TN woNVC, and diffusion metrics measures and topology organization changes revealed DTI showed extensive WM integrity alterations. However, above structural changes differed between TN wNVC and TN woNVC, and were related to specific chronic pain modulation mechanism.

**Conclusion:**

Abnormalities in characteristic regions of GM and WM structural network were found in TN woNVC compared with TN wNVC group, suggesting differences in pathophysiology of two types of TN.

## Introduction

Trigeminal neuralgia (TN), one of the most common pain conditions affect the craniofacial region, involves abnormalities in the peripheral and central trigeminal sensory pathways. The essential role of neurovascular compression (NVC) in classical TN pathophysiology has been supported by the efficiency of microvascular decompression (MVD) in treating classical TN since 1960s [1], and the NVC has been considered as “target” for the treatment of TN with NVC (TN wNVC) [2]. Accumulating evidence suggests that TN wNVC and TN without NVC (TN woNVC), classically similar forms of pain manifestations, may nevertheless differ in pathogenic mechanisms. Several studies have consistently reported that TN occurs and recurs in the absence of NVC [3–5], while NVC of the trigeminal nerve (TGN) exists in a sizeable population without TN [6]. There has been no recognized target for the treatment of TN wNVC, and patients with this type of TN have to underwent lesioning procedures, such as percutaneous balloon compression (PBC), percutaneous radiofrequency rhizotomy (PRR), or internal neurolysis (IN) [4, 7].

For some time, the abnormal neurovascular relationship in peripheral nerve system that trigger ascending changes in brain activity have been considered the foundation of TN wNVC pathophysiology and critical for the initiation and development of the pain attack events [8]. Studies have provided evidence that gray matter (GM) volume deficits and disruptions in the integrity of white matter (WM) have been observed in patients with CTN using structural and diffusion magnetic resonance imaging (MRI). According to a recent voxel-based morphometry (VBM) studies [9], the TN group showed significantly decreased gray matter volume in the bilateral superior/middle temporal gyrus (STG/MTG), bilateral parahippocampus, left anterior cingulate cortex (ACC), caudate nucleus, right fusiform gyrus, and right cerebellum compared with the HC. In addition to GM volume deficits, alterations of WM integrity in the thalamic-somatosensory tract ipsilateral to the site of NVC was reduced in CTN patients via topographical analysis [10].

The previous research of our team has revealed microstructural alteration of the trigeminal nerve root in TN woNVC via DTI [11], and we planned to further our understanding of the central mechanisms of TN woNVC. This study thus aimed to concurrently investigate GM and WM abnormalities in TN wNVC and TN woNVC, and to explore microstructual similarities and differences between the two subtypes, even to deduce the causality between trigeminal sensory abnormalities and structural brain changes, and identify promising novel targets for TN woNVC treatment. We performed voxel-based morphometry (VBM) and surface-based morphometry (SBM) of structural MRI data and applied graph theoretical analysis and group connectometry analysis based on DTI data retrieved from a consecutive cohort of patients with TN wNVC or TN woNVC and healthy controls (HC) in a single center database. To our knowledge, no previous study reports both alterations of GM volume and WM integrity in TN wNVC and TN woNVC patients, via VBM and SBM followed by diffusivities measures and graph theoretical analysis correlated to clinical covariates.

## Materials and methods

### Subjects

#### Inclusion and exclusion

##### Study participants and data-acquisition

This was a single-centre, retrospective study of patients treated surgically for CTN at Anhui Provincial Hospital between 2018 and 2020, approved by the Research Ethics Board of the Anhui Provincial Hospital. Diagnosis of CTN, defined as idiopathic, episodic, lancinating pain that lasted seconds, with pain-free episodes between attacks, by International Classification of Headache Disorders 3rd edition (ICHD-3) criteria, was the most important inclusion criteria. Potential study participants were identified from an operative database, and were included if they had undergone MVD or IN surgery performed by any one of two experienced neurosurgeons (Drs. X.-F.J. & M.W.), had proof of successful technical completion of surgery according to operative notes. Patients with secondary factors may resulting in TN, such as cranial tumors, multiple sclerosis (MS), or vertebrobasilar dolichoectasia resulting in brainstem compression, were excluded from this study [12]. Patients with a history of any previous non-TN neurosurgical procedures were excluded. Demographic and clinical data were obtained from physical and electronic patient charts. Patients were only included in the study once, even if they had undergone multiple TN procedures. In all cases, MRI images were collected within 1 week prior to the surgical procedure. Finally, a total of 23 patients with TN wNVC, 22 patients with TN wNVC and 45 HCs were prospectively included in our study.

#### Clinical characteristics and outcome assessment

The following demographic and clinical data were collected: sex, age at preoperative MRI, duration of TN, affected side, medications at time of preoperative MRI (carbamazepine or oxcarbazepine), and previous TN procedures. Duration of TN was defined as the amount of time between the dates of initial TN diagnosis to the date of preoperative brain MRI acquisition. Both the visual analog scale (VAS) and the short form of the McGill Pain Questionnaire (SF-MPQ) were applied for quantitative assessment of orofacial pain pre- and post-operatively, which represent the “sensory” and “affective” dimensions of pain severity respectively. Outcome were assessed from all patients in person at discharge (generally on postoperative day 7) and at the first outpatient visit (usually around postoperative day 50), which was used to calculate the average pain rating and obtain sensory, affective, and total pain indices.

### MRI data acquisition

All subjects underwent MRI using the following imaging protocol: sagittal-oriented 3D T2-weighted images (TR/TE = 2500.0/244.6 ms, field of view (FOV) = 256 × 256 mm^2^, with a 1 mm^3^ isotropic voxel size), sagittal-oriented 3D T1-weighted images (TR/TE = 8.6/4.0 ms, FOV = 256 × 256 mm^2^, 1 mm^3^ isotropic voxel size), and coronal-oriented 3D fluid-attenuated inversion recovery images (TR/TE = 4800.0/266.8 ms, FOV = 240 × 240 mm^2^, a 1 mm^3^ isotropic voxel size). In addition, DTI scans were obtained for all subjects. DTI was performed using spin-echo single shot echo-planar pulse sequences with a total of 32 different diffusion directions (TR/ TE = 8620/85 ms, FA = 90°, slice thickness = 2.25 mm, acquisition matrix = 120 × 120, FOV = 240 × 240 mm^2^, and b-value = 1000 s/ mm^2^). All of the scans were obtained using a 3T MRI scanner (Signa HDx; GE Healthcare; USA).

### T1 data processing

VBM was performed with CAT12, an SPM12 extension with the default pipeline (http://dbm.neuro.uni-jena.de/cat). This technique enables voxel-wise statistical analysis using the whole of automatically segmented gray and white matter. A unified segmentation approach was used. Subsequently, we used the affine registration algorithm to record all the native-space tissue segments to the standard Montreal Neurological Institute template (included in SPM12). The use of the exponentiated lie algebra toolbox (DARTEL) to all participants’ GM and WM was necessary to refine the inter-subject registration via the application of the diffeomorphic anatomical registration. Images were also modulated with the objectives of preserving gray matter data and minimizing distortion of normalization. Finally, an 8-mm full width at half maximum Gaussian filter was applied to allow statistical analysis.

Surface-based morphometry (SBM) were also performed with CAT12. This type of SBM analysis employs an algorithm for automatic cortical thickness and central surface estimation. Topological correction and spherical mapping for inter-subject alignment and spherical registration were performed. Cortical thickness was estimated using a projection-based methodology by calculating the distance between the inner (boundary between white and gray matter) and outer (boundary between gray matter and cerebrospinal fluid) cortical surfaces. Finally, thickness meshes were smoothed with a 15-mm Gaussian kernel, and a 20-mm kernel was used for the other parameters. The level of significance selected was uncorrected *p* < 0.001, with no cluster-forming height threshold.

### DTI data processing

Diffusivities measures were performed using DSI Studio (http://dsi-studio.labsolver.org). Fractional anisotropy (FA) measures directionality of water movement in brain tissues. The FA value was calculated using three eigenvalues of the diffusion tensor at each voxel. After creation of FA maps, the maps were smoothed (full-width half maximum = 8 mm). Then, we segmented GM and WM from standard space. In addition, to minimize registration errors, we applied an additional FA threshold to WM with FA values lower than 0.2. Finally, we compared FA and radial diffusivity (RD) values at each voxel in WM. FA is highly sensitive for detecting microstructural changes in the WM; RD is a putative marker of myelination, whereas an increased RD is suggestive of de- or dys- myelination [13].

Graph theoretical analysis was also performed using DSI Studio. Nodes were defined as anatomical regions, and edges were defined by fiber density. Graph theoretical analysis was performed as follows. First, a tractography was generated from the DTI data, which entails reading and parsing DICOM files, image reconstruction to characterize the major diffusion direction of the fiber, and fiber tracking. Afterwards, the connectivity matrix was generated, which was calculated from the count of the connecting tracts. The Automated Anatomical Labeling (AAL) template was used as the brain parcellation, and every white matter fiber was evaluated to determine its extreme points. This step included acquiring a whole brain fiber track, which placed the seeding at the whole brain level, spatial normalization, and definition of the regions of interest, and building the connectivity matrix. At last, we calculated the global graph theoretical network measures from the connectivity matrix, including the mean clustering coefficient, characteristic path length, small-worldness, global efficiency, and local efficiency, to obtain quantitative information regarding the global network properties. In addition, we also obtained measures of strength, betweenness centrality, and eigenvector centrality to investigate changes in hub organization.

### MVD and IN

Suboccipital retrosigmoid craniotomy and surgical corridor selection were performed in a manner previously described [14]. After complete exploration of the trigeminal root from the brainstem to the porus trigeminus of Meckel’s cave, and verifying the presence or absence of NVC of whole segment of TR in posterior fossa. The manipulation of MVD was performed in the situation of NVC [14], while internal neurolysis was performed in the situation of absence of NVC, by Drs. X.-F.J. and M.W. together, which had been published previously in detail by our team [15]. Both intraoperative findings and postoperative review of operative video were used to confirm the diagnosis of wNVC TN or woNVC TN prospectively.

### Statistical analyses

We employed two-sample *t*-tests (for age, sex, VAS, SF-MPQ) and chi-square tests (for gender, affected side, antiepileptic drugs) to examine individual differences. To determine statistical significance, we used analysis of covariance controlling for age, gender, education, drink, smoke, hypertension, and diabetes mellitus. For VBM and SBM studies, group comparisons between patients (all patients and those with and without NVC) and controls were performed using T contrasts to identify areas of increased and decreased abnormality, a *p*-value < 0.05 was considered significant for all calculations. In the FA and RD value comparison, the statistical threshold for significance was *p*-value < 0.005, uncorrected, and the minimum cluster size was set at 30 contiguous voxels. We investigated differences in the graph theoretical network measures between patients with TN and the HCs, comparisons were analyzed with a Student’s *t*-test; the statistical threshold for significance was *p*-value < 0.001. Statistical analysis was performed using the IBM SPSS Statistics® software (version 19.0).

## Results

### Demographic and clinical characteristics of subjects

Table 1 summarizes the demographic and clinical characteristics of the TN wNVC, TN woNVC and HC groups. There were no significant differences in age or sex among the 3 groups. In contrast to HCs, primary TN patients, displayed significantly higher VAS and SF-MPQ index scores; however, no significant differences in pain rating indices between TN wNVC and TN woNVC. Treatment dose of antiepileptic drugs was also not significantly difference between TN wNVC and TN woNVC groups.

**Table 1:** Demographic and clinical characteristics

Variables^a^	Healthy control (*N* = 45)	TN wNVC (*N* = 23)	TN woNVC (*N* = 22)	Statistics^b^
Age, y	49.36 ± 11.58	53.30 ± 8.66	47.77 ± 9.24	*F* = 1.74, *P* = 0.182
Sex (male / female), n	22/23	12/11	8/14	χ^2^ = 1.31, *P* = 0.520
Duration of illness, y	–	5.74 ± 3.35	4.97 ± 2.09	t = 0.92, *P* = 0.365
Side affected (L/R), n	–	9/14	10/12	χ^2^ = 0.18, *P* = 0.668
VAS pain rating	–	5.41 ± 2.06	4.48 ± 1.54	t = 1.65, *P* = 0.106
SF-MPQ-Total	–	13.13 ± 4.99	12.50 ± 4.19	t = 0.46, *P* = 0.650
SF-MPQ-Sensory	–	9.87 ± 4.45	6.77 ± 3.87	t = 2.48, *P* = 0.017
SF-MPQ-Affective	–	3.26 ± 2.36	5.72 ± 1.75	t = 3.97, *P* < 0.001
Antiepileptic medication				
Carbamazepine dose, mg/d	–	9.13 ± 5.24	6.13 ± 3.26	t = 2.29, *P* = 0.027

^a^Data are shown as mean ± SD or N

^b^ANOVA, chi-square test, or independent t test

### Neuroimaging findings

#### Grey matter volume changes (VBM)

**Fig. 1:**
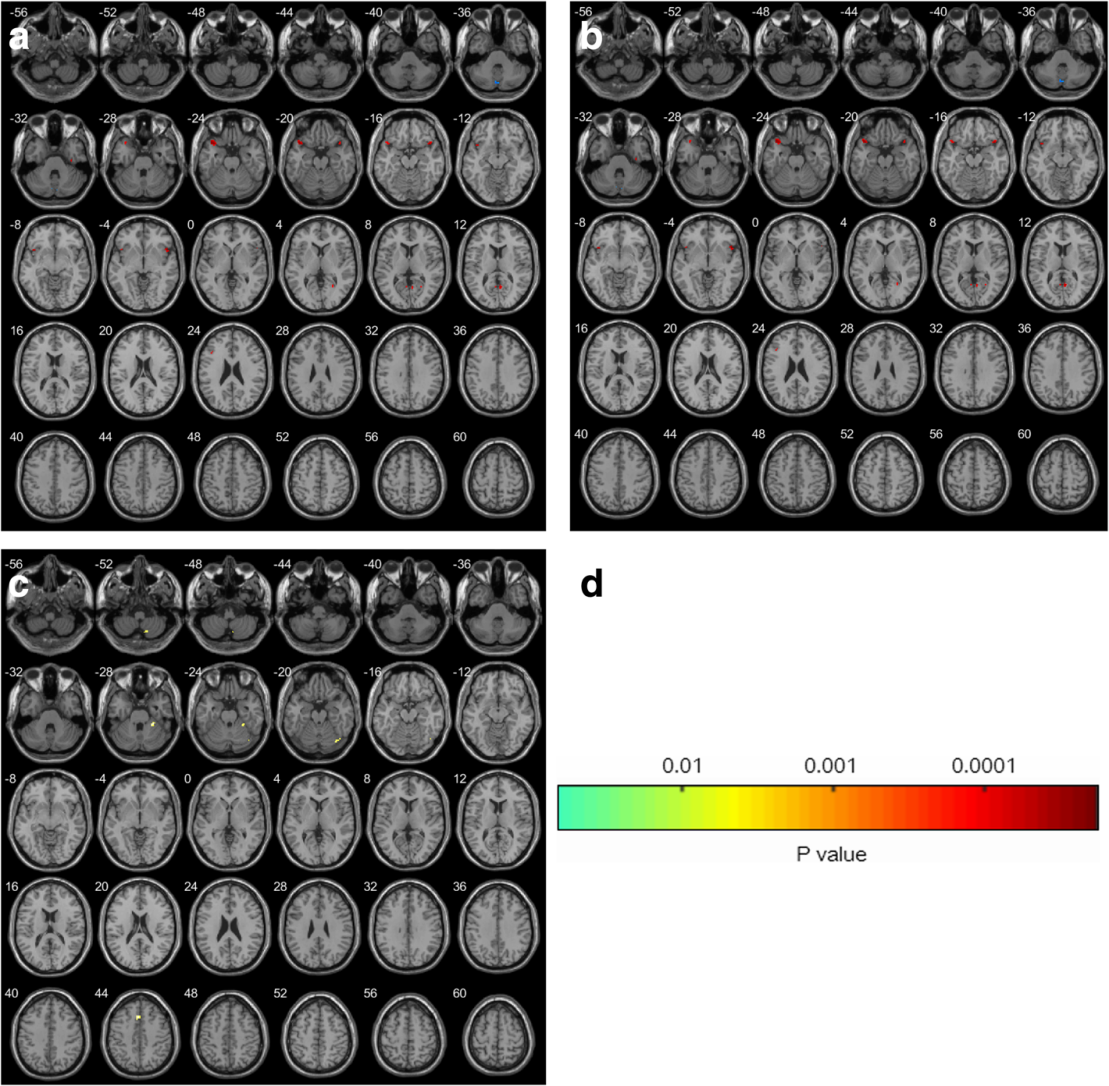
Results of the voxel-based morphometry analysis comparing 23 patients with TN wNVC and 22 patients with TN wNVC and 45 healthy controls. Results of the statistical analysis are overlaid on an axial T1 anatomical magnetic resonance imaging template

Comparison of grey matter volumes in HC and CTN revealed significant regional grey matter volume changes in several regions (Fig. 1, Table 2). GM volume deficits were explored between the TN wNVC and TN woNVC groups. Compared to HC, TN wNVC showed GM volume reductions in multiple cortical and subcortical areas, including the frontal (right inferior frontal gyrus and left superior frontal gyrus), temporal (bilateral middle temporal gyrus), and parietal (bilateral posterior parietal cortex, left angular gyrus) areas, as well as in the left thalamus and right cerebellum. Peak voxel was observed in the left thalamus (Fig. 1a). However, TN woNVC showed GM volume deficits in bilateral inferior temporal gyrus, bilateral posterior cingulate cortex (PCC), and moreover, their cluster sizes were relatively small. Taken together, TN wNVC groups showed extensively asymmetry GM volume deficits, while TN woNVC groups showed bilaterally symmetric GM volume deficits (Fig. 1b). In addition, a direct comparison between TN wNVC and TN woNVC revealed significant difference in right medial prefrontal cortex (mPFC) and left cerebellum (Fig. 1c).

**Table 2:** Pairwise comparisons of gray matter volume between TN wNVC, TN woNVC, and HC groups^a^

Anatomical region	Side	T	*P*	Peak coordinates (MNI)			Cluster size (mm^3^)
				x	y	z	
TN wNVC vs. HC							
Thalamus	L	5.45	0.017	−17	−21	−6	4430
Superior frontal gyrus	L	6.42	0.021	−28	45	41	1522
Inferior frontal gyrus	R	4.91	0.030	15	21	5	1733
Middle temporal gyrus	B	5.92	0.006	0	−36	14	1123
Posterior parietal cortex	B	5.03	0.027	0	−43	2	1893
Angular gyrus	L	4.55	0.045	−35	−37	11	961
Cerebellum	R	5.81	0.032	8	−38	−20	3206
TN woNVC vs. HC							
Inferior temporal gyrus	B	5.99	0.021	0	17	−23	2452
Posterior cingulate cortex	B	5.48	0.015	0	−14	10	1842
TN wNVC vs. TN woNVC							
Medial prefrontal cortex	R	6.83	0.019	2	9	44	1723
Cerebellum	L	5.88	0.034	−4	−21	−25	2109

That all MNI coordinates of maximum t values are selected in the significant region

That there was no significant difference in gray matter volume between the SZ and BD groups

*Abbreviations*: *SZ* Schizophrenia, *BD* Bipolar disorder, *HC* Healthy control, *MNI* Montreal Neurological Institute, *L* Left, *R* Right

^a^ Family-wise error corrected *P* < 0.017

#### Cortical thickness changes (SBM)

**Fig. 2:**
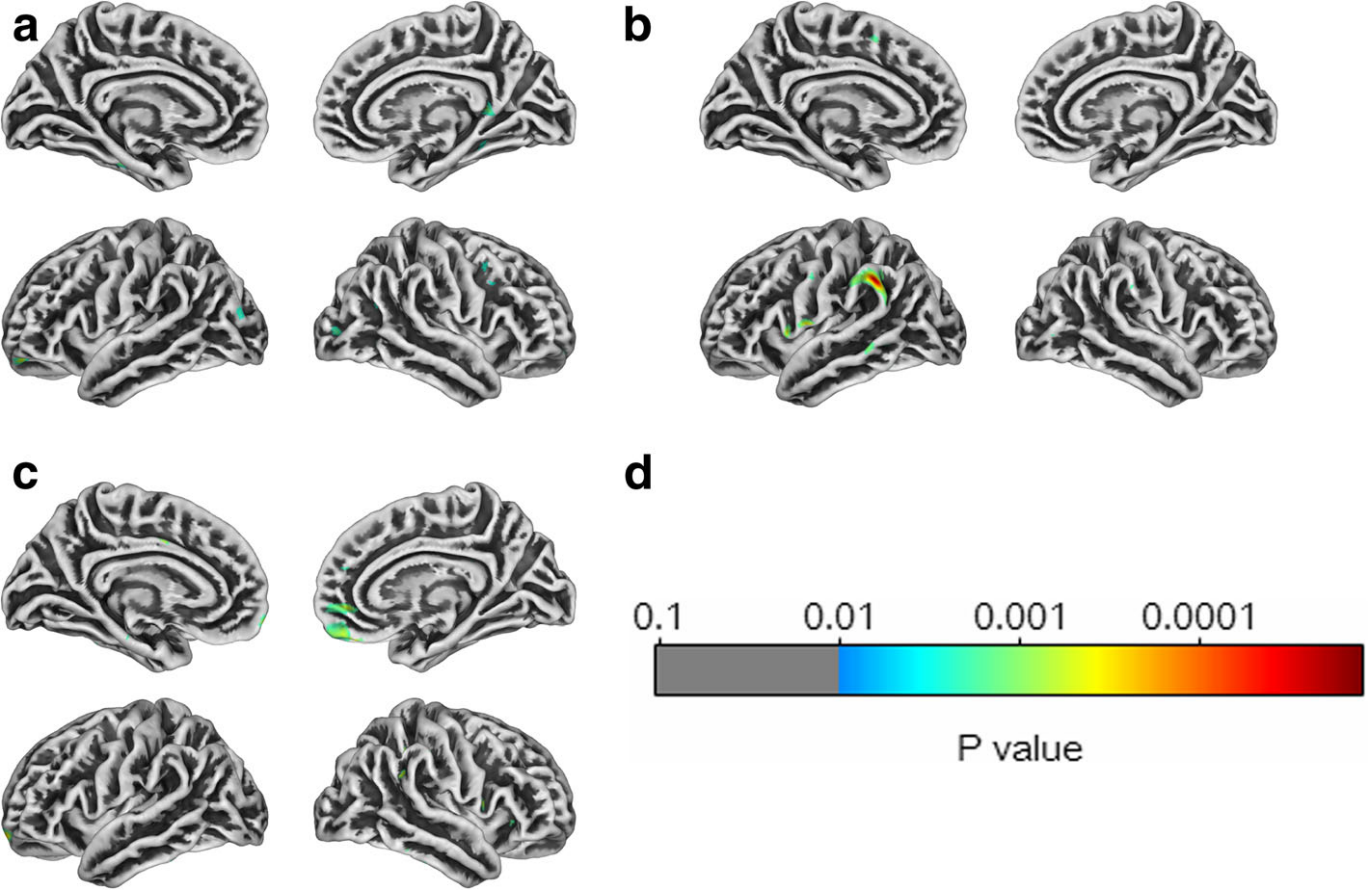
Results of the surface-based morphometry (cortical thickness) analysis comparing 23 patients with TN wNVC and 22 patients with TN wNVC and 45 healthy controls. Results of the statistical analysis (*p* map) are overlaid on a tridimensional rendering of the brain

In the comparison between TN wNVC and HC, three clusters of decreased cortical thickness were detected by SBM; localized to the left inferior frontal gyrus, right medial frontal gyrus, bilateral superior occipital gyrus and periaqueductal gray (PAG) matter (Fig. 2a). In the comparison between TN woNVC and HC, three cluster of decreased cortical thickness were detected, localized to the left angular gyrus, left medial frontal gyrus, left inferior temporal gyrus (Fig. 2b). However, in the direct comparison between TN wNVC and TN woNVC, two clusters of decreased cortical thickness were detected in TN woNVC, localized to bilateral mPFC (Fig. 2c) (Table 3).

**Table 3:** Pairwise comparisons of surface-based morphometry (SBM) analyses between TN wNVC, TN woNVC, and HC groups

Anatomical region	Side	T	*P*	Peak coordinates (MNI)			Cluster size (mm^3^)
				x	y	z	
TN wNVC vs. HC							
Inferior frontal gyrus	L	4.33	0.011	−12	25	8	1620
Medial frontal gyrus	R	4.36	0.032	21	17	24	1442
Superior occipital gyrus	B	3.94	0.028	0	−53	10	2371
TN woNVC vs. HC							
Angular gyrus	L	4.82	0.026	−36	−48	43	1363
Medial frontal gyrus	L	4.72	0.003	−12	45	12	1887
Inferior temporal gyrus	L	4.40	0.006	−27	−34	−14	1093
TN wNVC vs. TN woNVC							
Medial prefrontal cortex	B	4.95	0.010	0	36	−2	1989

#### Diffusion value changes (DTI)

**Fig. 3:**
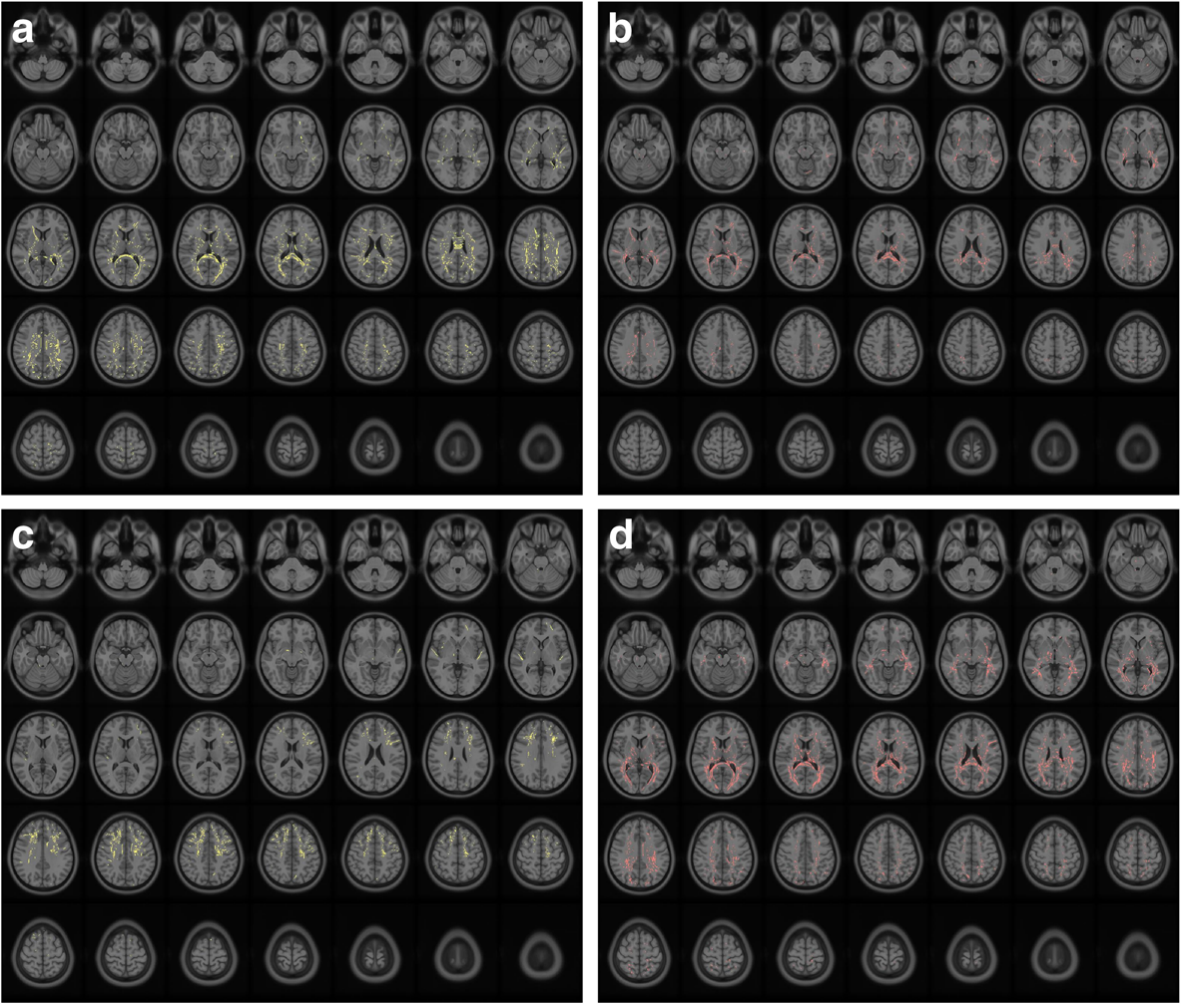
Results of the diffusivities measures (fractional anisotropy and radial diffusivity) analysis comparing 23 patients with TN wNVC and 22 patients with TN wNVC and 45 healthy controls. Results of the statistical analysis are overlaid on an axial T1 anatomical magnetic resonance imaging template

Disruptions of WM integrity were explored in the TN wNVC and TN woNVC groups. Overall, wide-spread alterations in diffusion were identified in both the TN wNVC and TN woNVC groups (Tables 4 and 5). Compared to HC, patients with TN wNVC showed FA decreased in bilateral superior corona radiata, right anterior limb of internal capsule, splenium of corpus callosum, body of corpus callosum, right cingulum, and left genu of internal capsule, RD increased in the splenium of the corpus callosum, right anterior limb of internal capsule, left posterior thalamic radiation, and bilateral posterior corona radiata. (Fig. 3a, b) However, patients with TN woNVC showed FA decreased in the bilateral anterior corona radiata, and bilateral fasciculus uncinatus, and RD increased in the splenium of corpus callosum, right anterior thalamic radiation, left anterior limb of internal, left external capsule and bilateral superior corona radiata. Further, direct comparisons of DTI parameters between TN wNVC and TN woNVC revealed no significant differences (Fig. 3c, d).

**Table 4:** Comparisons of diffusion indexes between TN wNVC and HC groups

Anatomical region	Side	T max	*P*	Peak coordinates (MNI)			Cluster size (mm)
				x	y	z	
FA TN wNVC vs. HC							
Superior corona radiata	L	4.124	0.0022	−31	−30	27	218
Superior corona radiata	R	4.392	0.0028	28	−15	33	304
Anterior limb of internal capsule	R	5.920	0.0039	24	26	4	412
Splenium of corpus callosum		4.729	0.0030	−2	−36	31	542
Body of corpus callosum		5.325	0.0043	5	11	19	1332
Cingulum	R	4.782	0.0036	8	14	32	392
Genu of internal capsule	L	3.882	0.0019	−18	21	13	430
RD TN wNVC vs. HC							
Splenium of corpus callosum		4.644	0.0037	3	−29	37	526
Anterior limb of internal capsule	R	5.801	0.0040	22	30	8	669
Posterior thalamic radiation	L	5.261	0.0035	−27	−49	21	573
Posterior corona radiata	L	4.602	0.0030	−37	−52	31	629
Posterior corona radiata	R	3.772	0.0017	28	−44	29	478

**Table 5:** Comparisons of diffusion indexes between TN woNVC and HC groups

Anatomical region	Side	T max	Peak coordinates (MNI)			Cluster size (mm)
			x	y	z	
FA TN woNVC vs. HC						
Anterior corona radiata	L	5.82	−31	45	32	662
Anterior corona radiata	R	5.91	27	47	30	732
Fasciculus uncinatus	L	4.23	−42	9	−5	403
Fasciculus uncinatus	R	4.05	38	11	−9	437
RD TN woNVC vs. HC						
Splenium of corpus callosum		4.665	3	−29	37	530
Anterior thalamic radiation	R	5.023	5	−10	14	572
Anterior limb of internal capsule	L	4.032	−27	31	16	539
External capsule	L	3.782	−33	26	23	402
Superior corona radiata	L	5.820	−22	8	43	659
Superior corona radiata	R	5.521	16	7	37	587

#### Topology and hubs organization changes (DTI)

The measures of global topology in patients with TN wNVC or TN woNVC showed different characteristics, compared with that in HC. As compared with the HC group, patients with TN wNVC had significant lower global efficiency, local efficiency and higher characteristic path length, while patients with TN woNVC had significant higher global efficiency, local efficiency, and lower small-worldness (Table 6). There were significant differences in the reorganization of hubs organization in both two groups of patients compared to healthy subjects. In the comparison between TN wNVC and HC, the strength of left middle frontal gyrus, left inferior occipital gyrus, right posterior parietal cortex and left angular gyrus in the patients with TN wNVC were lower than those in healthy subjects. In addition, the betweenness centrality of right middle temporal gyrus, right insula, left angular gyrus and left thalamus in the patients with TN wNVC were lower than those in HC. Moreover, the eigenvector centrality of left angular gyrus, right superior frontal gyrus, left posterior parietal gyrus and left middle frontal gyrus in the patients with TN wNVC were lower than those in healthy subjects. In the comparison between TN woNVC and HC, the strength of left inferior temporal gyrus, right posterior cingulate cortex, right hippocampus, left superior temporal gyrus and right inferior frontal gyrus in the patients with TN woNVC were lower than those in HC. Besides, the betweenness centrality of left inferior frontal gyrus, left hippocampus, left inferior temporal gyrus and right posterior cingulate cortex in the patients with TN woNVC were lower than those in HC. Moreover, the eigenvector centrality of left posterior cingulate cortex, right posterior cingulate cortex and left angular gyrus in the patients with TN woNVC were lower than those in HC.

**Table 6:** Comparison of global topographic parameters between TN wNVC and TN woNVC compared with HC

Network measures	TN wNVC		TN woNVC		HC
	Mean ± SD	*p*-value	Mean ± SD	*p*-value	
Mean clustering coefficient	0.2867 ± 0.0085	0.3486	0.2883 ± 0.0087	0.9256	0.2881 ± 0.0041
Characteristic path length	4.2004 ± 0.2313	0.0022	3.6647 ± 0.5478	0.2681	3.8229 ± 0.5431
Small-worldness	0.1891 ± 0.0076	0.1093	0.1825 ± 0.0135	0.0009	0.1932 ± 0.0108
Global efficiency	0.7954 ± 0.1112	0.0068	0.9259 ± 0.1101	0.0027	0.8570 ± 0.0699
Local efficiency	1.1535 ± 0.1615	0.0235	1.7174 ± 0.5502	0.0013	1.3385 ± 0.3635

### Intraoperative findings

The absence or the presence of NVC was verified in a number of 21 patients respectively after complete exploration of the trigeminal root from the porus of the trigeminal Meckel’s cave to the trigeminal root entry zone (TREZ) at the pons. The intraoperative findings were recorded and contributed to the diagnosis of TN wNVC and TN woNVC, combined with preoperative clinical assessment.

## Discussion

The current study is to investigate the associations and differences between variations in brain GM structure (GM volume and cortical thickness), WM integrity (i.e., DTI parameters, global graph theoretical network measures, and hubs organization) and the factor of NVC within a cohort of patients with TN and sex-, age- matched HC. Overall, the results obtained are in line with the concept that TN is associated with GM and WM abnormalities [16], while, there are obviously different manifestations between TN wNVC and TN woNVC. To our knowledge, this is the first study to analyze brain pathophysiology and pathogenetic mechanism in TN wNVC and TN woNVC, via structural and diffusion MRI.

### GM alterations occur in common areas in TN wNVC and TN woNVC but are more widespread in TN woNVC

In this study, compared to HC, in TN wNVC, the GM volume deficits were widely distributed across the thalamus, superior frontal gyrus, inferior frontal gyrus, middle temporal gyrus, angular gyrus, and posterior central gyrus. These affected regions were mostly consistent with those reported by previously published large-sized VBM analyses and meta-analyses which have reported that patients with CTN show GM volume deficits throughout the frontal, temporal and parietal lobes; cingulate and insular cortices; and thalamus [17–23]. In contrast, in patients with TN woNVC as compared with HC, we observed almost all reductions in GM volume were bilateral symmetrical, which were limited to the bilateral inferior temporal gyrus, bilateral PCC. Overall, our VBM analyses could draw the conclusion that TN wNVC and TN woNVC display quite different types of GM volume deficits when compared with HC group, although share common deficits in the posterior cingulate cortex and inferior temporal gyrus. Meanwhile, in the cortical thickness analysis, left angular gyrus was detected in the comparison between TN woNVC and HC, and right mPFC was significantly decreased in TN woNVC when compared with TN wNVC. Medial temporal lobe plays a role in sensitization-like processes related to the affective component of pain, which may be linked to impaired memory in fibromyalgia [24]. The association among TN, migraine, cluster headache, and low back pain in epidemiology and pathogenesis has been acknowledged [25–27]. Thalamic-somatosensory dysfunction and dysrhythmia have been proved to be associated with TN, migraine and low back pain, and the abnormal functional connectivity between the thalamus and attentional cerebral networks has been substantially proved at rest during migraine attacks [10, 28–31]. With regard to the PCC, it play an important role in pain modulation, and has been considered as crucial brain regions of default mode network (DMN) [32]. Moreover, the direct comparison between TN wNVC and TN woNVC showed both the GM volume and cortical thickness of right MPC were decreased in TN woNVC. The mPFC is one of the crucial component in conditioned pain modulation (CPM), especially participating in top-down pain processing [33, 34]. The mPFC also compose the DMN with PCC, angular gyrus [35], and the TN woNVC can be associated with the changes of these structure according to our findings.

Recent series of studies have identified several cortical and subcortical regions associated with GM volume reduction in patients with TN wNVC [17, 19, 20, 22, 23, 36, 37]. Putting the findings of these studies together, it can be inferred that TN wNVC is associated with extensive deficits. Our VBM and SBM analyses performed simultaneous comparisons between TN wNVC vs. HC and between TN woNVC vs. HC and confirmed that GM volume reductions are typically more extensive in TN wNVC than in TN woNVC, and that there is an overlap in the affected regions between the two disorders. In addition, patterns of the overlap between patients with TN wNVC and those with TN woNVC suggest that GM volume and cortical thickness deficits were focused in the medial frontal gyrus, which might be common neurobiological substrates across TN wNVC than in TN woNVC. Finally, in the comparison of GM between TN wNVC and TN woNVC, patients with TN wNVC showed a widespread reduction in GM volume, including insula, somatosensory cortex, and thalamus, which was associated with bottom-up pain processing areas [38]; whereas those with TN woNVC showed limited deficits, such as the PFC, might be associated with the top-down pain processing [39].

### Widespread compromise of WM integrity occurs in both TN wNVC and TN woNVC

A combined interpretation of these DTI parameters may allow us to better characterize the alterations of WM microstructure in TN wNVC and TN woNVC. We observed that compared to HCs, significant decreases in FA and increases in RD were widespread over areas of the whole-brain WM skeleton in both TN wNVC and TN woNVC. Regarding TN wNVC, decreases in FA were coupled with increases in RD in areas of the corpus callosum, corona radiata and internal capsule, which indicates probable damage to pain integration, cognitive-affective, and motor functions, also could be relative to compensatory mechanisms for this type of TN [40, 41]. In addition, increases in RD in the areas of the posterior thalamic radiation, which is suggestive of damage to the microstructural properties of the thalamic-somatosensory. However, TN woNVC showed decreases in FA were located to bilaterally symmetrically in anterior corona radiata and fasciculus uncinatus, while increases in RD in the areas of thalamic radiation, internal capsule, external capsule, corpus callosum and bilateral superior corona radiata. Overall, our findings related to TN wNVC and TN woNVC are in accordance with the results of a recent review of DTI data which showed significant global alterations in corpus callosum, cingulum bundle, coronal radiata, and superior longitudinal fasciculus [16].

Further, in order to investigate the effect of WM tissue alterations on the structural brain network, and reveal insight into potential pathophysiological mechanisms underlying TN at the network level, we applied graph theory to obtain topographic parameters. Brain structural connectivity via graph theory analysis has been demonstrated effective for predicting brain functional complexity of BOLD-fMRI signals [42]. With the results of network analysis on a global scale, different patterns of abnormal connectivity in TN wNVC and TN woNVC were found. In TN wNVC, the brain connections of temporal gyrus, occipital lobe, insula, and thalamus are related to multisensory integration of auditory, visual, olfactory, and somatosensory information [43]. Altered connectivity in these regions may interfere the temporal filtering of nociceptive information in offset analgesia [39], and underlie the psychological disturbances including anxiety, fear, and panic in TN patients [41], and sensory, cognitive-affective, and modulatory aspects of pain [30]. However, patients with TN woNVC exhibited higher local network integration in the hippocampus, PFC and angular gyrus as compared to HC, which may undermine the spatial filtering of nociceptive information in CPM, especially the top-down pain processing [39]. We inferred that such changes in network connectivity may be an initial key step in the process that ultimately results in clinical symptoms. Our findings suggest that even without the influence of NVC, TN woNVC may induce alterations in anatomical brain connections, possibly due to alteration of top-down pain processing.

There are several methodological limitations to this study. First, we only investigated 23 patients with TN wNVC and 22 patients with TN wNVC, larger sample sizes may be needed in our future study. Second, further study of the functional connectivity and brain white matter plasticity alterations of TN woNVC still need to be complete in order to verify the result of structural network in this research.

## Conclusion

Overall, our findings suggest that TN is associated with anatomical changes within various brain structures involved in pain modulation. Whilst our data clearly show that, compared with TN wNVC group, abnormalities in characteristic regions of GM and WM structural network were found in TN woNVC group. Evidence of altered processing CPM raises the possibility of dynamic changes in top-down pain processing areas, which may either trigger or alter the sensitivity of the brain so that an external trigger results in a trigeminal neuralgia attack without the participation of NVC. Future investigations will explore the resting state of brains of TN woNVC, if dynamic changes in brain function overlap certain regions of structural alterations, it is possible to identify promising novel target for we may be in a position to modify these changes and potentially prevent the triggering of TN woNVC.

## Abbreviations

BOLD: Blood oxygen level-dependent
CPM: Conditioned pain modulation
DTI: Diffusion tensor imaging
FA: Fraction of anisotropy
HC: Healthy control
IN: Internal neurolysis
MRI: Magnetic resonance imaging
MVD: Microvascular decompression
NVC: Neurovascular compression
RD: Radial diffusivity
SBM: Surface-based morphometry
SF-MPQ: Short-form McGill Pain Questionnaire
TN: Trigeminal neuralgia
TN wNVC: Trigeminal neuralgia with neurovascular compression
TN woNVC: Trigeminal neuralgia without neurovascular compression
VAS: Visual analog scale
VBM: Voxel-based morphometry
